# Protective effect of vitamin E on cypermethrin-induced follicular atresia in rat ovary: Evidence for energy dependent mechanism

**Published:** 2016-06-15

**Authors:** Morteza Molavi, Mazdak Razi, Hadi Cheraghi, Mona Khorramjouy, Araz Ostadi, Safa Gholirad

**Affiliations:** 1 *Private Veterinary Practitioner, Urmia, Iran;*; 2 *Department of Basic Sciences, Faculty of Veterinary Medicine, Urmia University, Urmia, Iran;*; 3 *Department of Clinical Pathology, Faculty of Veterinary Medicine, University of Tehran, Tehran, Iran;*; 4 *Department of Pharmacology and Toxicology, Faculty of Pharmacy, Shahid Beheshti University of Medical Sciences, Tehran, Iran.*

**Keywords:** Apoptosis, Caspase-3, Cypermethrin, Follicular atresia, GLUT-1

## Abstract

It has been shown that chronic exposure to cypermethrin (CPM), a pyrethroid pesticide, results in follicular atresia via pathologically affecting angiogenesis, disrupting endocrine potential and enhancing oxidative stress. This study was aimed to uncover the CPM-exposed energy dependent follicular cells apoptosis and to estimate protective effect of vitamin E (VitE) as a potent antioxidant. Thirty six Wistar rats were divided into six groups (n = 6 rats for each group) including; control-sham, CPM-received (CPM, 75 mg kg^-1^, intraperitoneally), and CPM and VitE-treated (VitE, 150 mg kg^-1^, orally) for 14 and 24 days. The protein biosynthesis of glucose transporter-1 (GLUT-1) and caspase-3 in follicles were estimated by using immuno-histochemical staining at preantral and antral stages. Moreover, the periodic acid Schiff (PAS) staining was performed in order to evaluate the intracytoplasmic carbohydrate ratio in follicular cells and oocyte. Percentages of follicles with GLUT-1, Caspase-3 and PAS-positive cells were compared between groups. Immunohistochemical analyses showed that, VitE significantly up-regulated the GLUT-1 expression and improved the intracytoplasmic carbohydrate supplementation especially at preantral follicles. The cross sections from the CPM-exposed ovaries represented remarkable elevation in percentage of atretic preantral and antral follicles with caspase-3 biosynthesis, which was remarkably (*p* < 0.05) diminished in VitE co-treated groups. In conclusion, our data showed that VitE by up-regulating of the GLUT-1 biosynthesis improved glucose uptake at follicular cells and oocyte levels that in turn inhibited pro-apoptotic protein caspase-3 biosynthesis.

## Introduction

Cypermethrin (CMP), cyano-(3-phenoxyphenyl) methyl 3-(2,2-dichloroethenyl)-2,2-dimethylcyclopropane-1-carbo-xylate, a pyrethroid pesticide, is widely used against pests in agriculture and veterinary medicine also for controlling pests at homes worldwide.^1^ Previous studies illustrated that pyrethroids possess hormonal interactions, classified as endocrine disruptors and focused on the pyrethroids-induced damages on gonadal hormone levels.^2^^,^^3^ On the other hand, it has been suggested pyrethroid pesticides are associated with certain male reproductive damages.^4^^-^^6^ Other reports have demonstrated further detrimental effects following dermal exposure to CPM such as; numbness, tingling, itching, irritation, urinary incontinence , incoordination, seizures and death.^7^

Recently, Molavi *et al* has shown that CMP, impacts the ovarian follicular growth by affecting gonadotropins, follicular stimulating hormone (FSH) and luteinization hormone (LH) biosynthesis. Furthermore, CPM disrupted endocrine system, induced biochemical impairments (oxidative stress), enhanced the expression of p53 and promoted follicular atresia.^8^ The oocyte apoptosis is considered as the main cause of follicular atresia at early preantral stages.^9^^-^^12^ Whereas, the granulosa cells apoptosis, degeneration and necrosis as germ cells eliminations are estimated as main reasons for atresia at later antral stages.^13^^,^^14^

In mammal cells, caspase-3 is a frequently activated death protease in granulosa cells at later pre-ovulatory follicles.^15^ Yacobi *et al.* showed that gonadotropins play an essential role in inhibiting pro-caspase-3, pro-caspase-7 and even caspase-3 and caspase-7 biosynthesis. Accordingly, surcharging these hormones in *in vitro* resulted in decreased granulosa cells apoptosis.^16^ Therefore, gonadotropins by promoting biosynthesis of survival proteins control the follicular cells apoptosis further to caspase-3 biosynthesis.^17^^,^^18^

Glucose uptake is mediated by a number of facilitative sugar transporters. Until now, 13 facilitative sugar transporters (GLUT1-12 and HMIT) have been recognized, which exhibit different substrate specificities, kinetic properties as well as tissue expression/biosynthesis profiles.^19^ Among these variations, the GLUT-1, 3 and 4 have been reported to be expressed in the ovary of sheep^20^, rat^21^^,^^22^ and mouse.^23^ Kodaman and Behrman showed that co-incubating the granulosa cells with FSH and insulin dependent growth factor (IGF-1) up-regulates the mRNA and protein expressions of GLUT-1 in rat's granulosa cells. This finding suggests that the stimulatory effects of FSH and IGF-1 on dehydroascorbic acid (DHAA) transport are mediated with GLUT-1 and GLUT-4 in the granulosa cells.^22^ On the other hand, Zhang *et al. *showed that FSH affects glucose uptake and induces ovarian development by increasing GLUT gene expression and/or affecting GLUT localization/translocation.^24^ As ovaries are highly dynamic organs, the carbohydrates play essential roles in accelerating the biochemical, metabolic and physiological pathways in cells and/or between cells. 

Vitamin E (VitE) is a vital component of biological membranes with an antioxidant function,^9^^,^^10^ which involves in protecting membrane stability against free radicals-induced peroxidation.^9^^-^^12^ It appears to be the first line of defense faces free radicals and protects the cell from oxidants-induced damages.^8^

To understand the importance of this impairment, one should note that most of structural and functional proteins are placed on and/or in the cell membrane including GLUTs. Considering the distinctive impact of the reactive oxygen species (ROS) on lipids and proteins via peroxodation, administration of potential antioxidants prevents these derangements. Indeed, ROS are reported to be induced by pyrethroids, especially CPM.^25^

## Materials and Methods

**Chemicals. **Periodic acid-Schiff (PAS) staining kit purchased from Asia Chem Co. (Sari, Iran). The rabbit anti-mouse primary antibody for GLUT-1 (Biocare Co., Birmingham, UK) was purchased from Life Teb Gen Co. (Tehran, Iran). The rabbit anti-mouse primary antibody for caspase-3 (Gennova Scientific S.L. Seville, Spain) was assigned from Pishtaz Teb Co. (Tehran, Iran).

**Animals and study design. **Laboratory Wistar albino rats (10 to 12 weeks old), with body weight of 180 to 200 g were used. The animals were housed under controlled condition of illumination (12 hr light/12 hr dark cycle) and temperature 20 to 25 ˚C throughout the experiment period. Standard pellet diet and water were provided* ad libitum*. Both the international guidelines for the animal’s welfare and the compatible local regulations for experiments were respected during the study. The rats were randomly divided into six groups of six animals in each experimental and control: Groups 1 and 2, (control-sham) received saline (2 mL kg^-1^, orally per day). Group 3 received CPM (75 mg kg^-1^, 1/4 of LD_50_) for 14 days. Group 4 was administrated with the same dose of CPM for 24 days. In group 5, CPM was co-administrated with VitE (150 mg kg^-1^) for 14 days.^8^ Groups 6 received the same schedule as group 5 for 24 days. Following 14 and 24 days, the rats were anesthetized with 70 mg kg^-1^ ketamine (Alfasan, Woerden, The Netherlands) and 5 mg kg^-1 ^xylazine (Alfasan). Then, the animals were euthanized with special CO_2_ devise (ADACO, Iran) and the tissue samples including the right and left ovaries were dissected out and fixed in 10% formalin (Mojallali Chemical Complex, Tehran, Iran). 

**Immunohistochemical (IHC) staining. **Tissue section slides were heated at 60 ˚C for approximately 25 min in a hot air oven (Venticell; MMM, Einrichtungen, Germany). The tissue sections were de-paraffinized in xylene and rehydrated using an alcohol gradient. The antigen retrieval process was performed in 10 mM sodium citrate buffer. Immunohistochemical staining was conducted according to the manufacturer's protocol (Biocare Medical, Concord, USA and Gennova Scientific S.L. Seville, Spain). Briefly, endogenous peroxidase was blocked in a peroxidase blocking solution (0.03% hydrogen peroxide containing sodium acid) for 5 min. Tissue sections were washed gently with washing buffer and subsequently incubated with GLUT-1 (1:500) and caspase-3 biotinylated primary antibodies for 15 min. The sections were rinsed gently with washing buffer and placed in a buffer bath. The slides were then placed in a humidified chamber with a sufficient amount of streptavidin conjugated to horse-radish peroxidase (HRP) in phosphate-buffered saline (PBS) containing an anti-microbial agent. The slides were incubated for 15 min. Subsequently, the tissue sections were rinsed gently in washing buffer and placed in a buffer bath. A 3,3'-diaminobenzidine (DAB) chromogen was added to the tissue sections and incubated for 5 min, followed by washing and counter staining with hema-toxylin for 5 sec. The sections were then dipped in weak ammonia (0.037 Mol L^-1^) 10X, rinsed with distilled water and cover slipped. Positive immunohistochemical staining was observed as brown stains under a light microscope. ^26^

**Immunohistochemical (IHC) staining. **Tissue section slides were heated at 60 ˚C for approximately 25 min in a hot air oven (Venticell; MMM, Einrichtungen, Germany). The tissue sections were de-paraffinized in xylene and rehydrated using an alcohol gradient. The antigen retrieval process was performed in 10 mM sodium citrate buffer. Immunohistochemical staining was conducted according to the manufacturer's protocol (Biocare Medical, Concord, USA and Gennova Scientific S.L. Seville, Spain). Briefly, endogenous peroxidase was blocked in a peroxidase blocking solution (0.03% hydrogen peroxide containing sodium acid) for 5 min. Tissue sections were washed gently with washing buffer and subsequently incubated with GLUT-1 (1:500) and caspase-3 biotinylated primary antibodies for 15 min. The sections were rinsed gently with washing buffer and placed in a buffer bath. The slides were then placed in a humidified chamber with a sufficient amount of streptavidin conjugated to horse-radish peroxidase (HRP) in phosphate-buffered saline (PBS) containing an anti-microbial agent. The slides were incubated for 15 min. Subsequently, the tissue sections were rinsed gently in washing buffer and placed in a buffer bath. A 3,3'-diaminobenzidine (DAB) chromogen was added to the tissue sections and incubated for 5 min, followed by washing and counter staining with hema-toxylin for 5 sec. The sections were then dipped in weak ammonia (0.037 Mol L^-1^) 10X, rinsed with distilled water and cover slipped. Positive immunohistochemical staining was observed as brown stains under a light microscope. ^26^

**Assessment of intracytoplasmic carbohydrate ratio. **To evaluate the carbohydrate ratio, the PAS staining technique was employed. The staining was conducted based on manufacture’s protocol. In brief, the paraffin sectioned specimens were deparraffinized and hydrated. The hydrated slides were oxidized in 5% periodic acid solution for 5 min. After rinsing in distilled water, the slides were placed in Schiff reagent for 15 min before being washed with lukewarm water. After 5 min, the sections were incubated with Borax’s solution and then counterstained with Meyer’s hematoxylin. 

**Computer assisted imaging and analyses. **Distribution of the cells with positive reaction for chromogen in two different IHC staining of GLUT-1 and caspase-3 was assessed by using Image Pro-Insight software (version 9.00; Cybermetric Co., Phoenix, USA). Moreover, the reactions for PAS staining were classified as faint, moderate and intensive. Axiovision Rel software (version 4.8, Carl Zeiss AG, USA) calibrated camera (Sony Corp., Tokyo, Japan) was used to estimate the PAS-positive cells distribution. 

**Comparing histological findings between groups. **For quantitative data assessment, mean percentages of follicles at preantral and antral stages, which exhibited GLUT-1 and casapase-3 were histologically evaluated and compared between groups, statistically. The percentages of follicles with intact intracytoplasmic carbohydrate were estimated and compared between groups. 

**Statistical analyses. **Quantitative data were statistically analyzed by using SPSS software (version 16.0; IBM, Armonk, USA) and *p* < 0.05 was considered as significant difference. One way analysis of variance was used to compare the mean percentage of follicles with specific histological features. All data were presented as mean ± standard deviation.

## Results

**Fig. 1:**
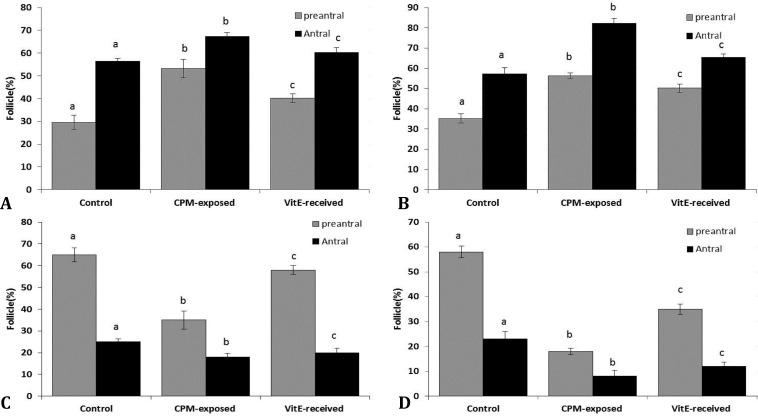
Mean percentage of follicles with caspase-3 and GLUT-1 expression: **A**) day 14 and **B**) day 24 after exposure to CPM for showing caspase-3 expression; **C**) day 14 and **D**) day 24 after exposure to CPM for showing GLUT-1 expression, all data are presented as Mean ± SD

^abc^ represent significant differences between marked data (*p* < 0.05).

Histological analyses showed that, animals in CPM-exposed group exhibited a significant enhancement in the caspase-3 protein expression. Accordingly, higher percentages of follicles with caspase-3 positive cells were revealed in CPM-exposed groups. Comparing groups based on exposure time against CPM, showed remarkable increase of the atretic follicles with caspase-3 expression (Figs. 1A and 1B).

**Fig. 2:**
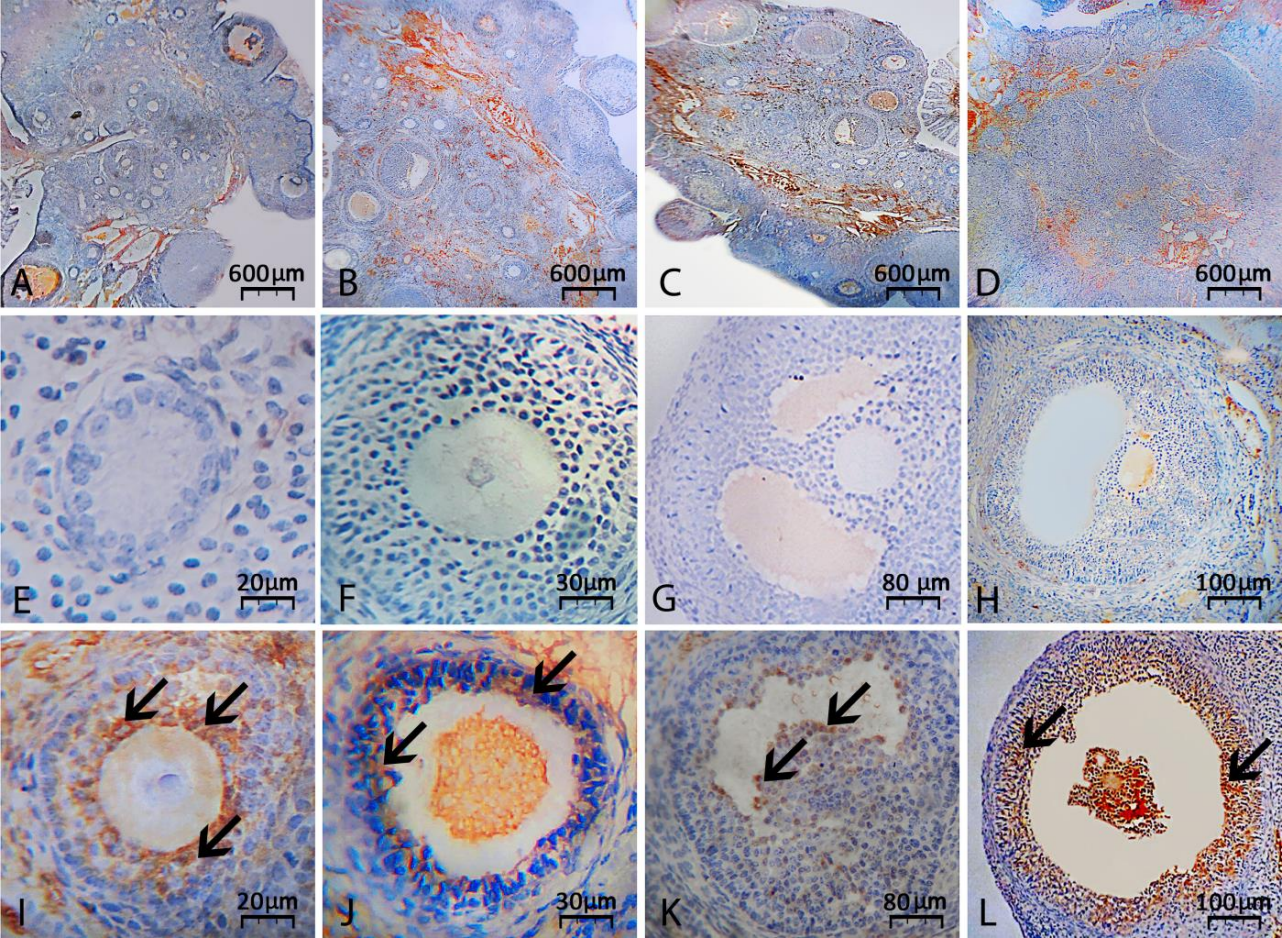
Immunohistochemical staining for caspase-3: (**A**) Vitamin E-received group on day 14, (**B**) CPM alone-exposed group on day 14, (**C**) Vitamin E-received group on day 24 and (**D**) CMP alone-exposed group on day 24 after exposure. Co-administrating of vitamin E significantly reduced biosynthesis of caspase-3 protein on days 14 and 24 after exposure to CPM. Second row is representing intact early secondary (**E**), late secondary (**F**), tertiary (**G**) and graafian (**H**) follicles, which are not expressing caspase-3. However, the atretic early secondary (**I**), late secondary (**J**), tertiary (**K**) and graafian (**L**) follicles are representing intensive caspase-3 expression. Arrows are representing chromogens for caspase-3, (IHC

However, VitE administration resulted in a remarkable reduction in CPM-induced caspase-3 over-expression after both 14 and 24 days exposure (*p* < 0.05), (Fig. 2). 

Immunohistochemical staining for GLUT-1 showed a significant reduction in percentage of follicles with GLUT-1 stained follicular cells in CPM-exposed groups (Figs. 1C and 1D). Whereas, the cross sections from the ovaries of animals in VitE-treated groups showed a considerable amelioration.

**Fig. 3:**
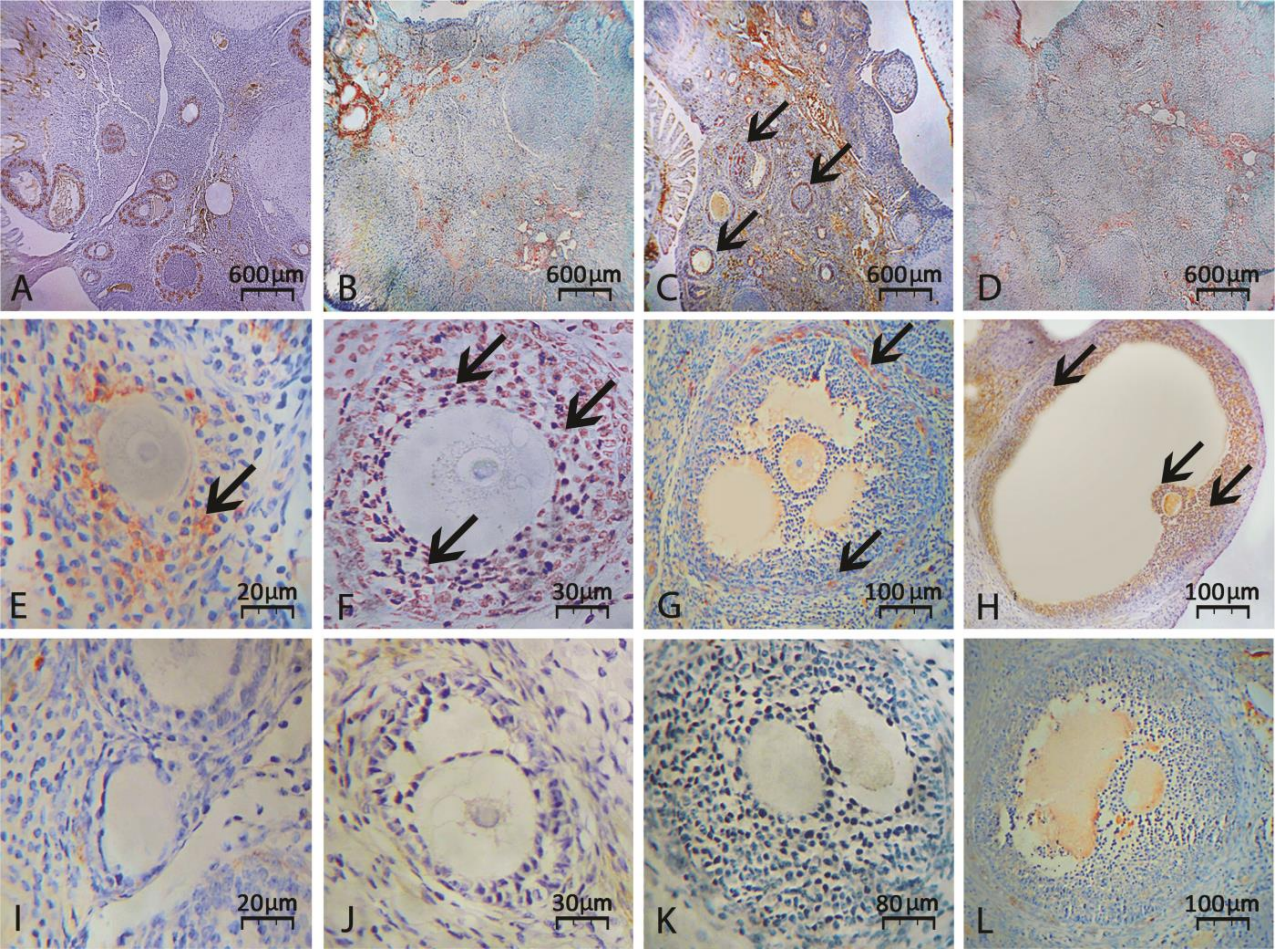
Immunohistochemical staining for GLUT-1, (**A**) Vitamin E-received group on day 14, (**B**) CPM alone-exposed group on day 14, (**C**) Vitamin E-received group on day 24 and (**D**) CMP alone-exposed group on day 24 after exposure. Note decreased reaction for GLUT-1 in CPM alone-exposed groups, which is significantly increased in VitE-received cross sections. Intact follicles at primary, secondary and tertiary stages and graafian follicle are presented in second row in figures (**E**), (**F**), (**G**) and (**H**), respectively. Note atretic follicles with no and/or low biosynthesis of GLUT-1 in last row as primary (**I**), secondary (**J**), tertiary (**K**) and graafian (**L**) follicles. As marked with arrows, intact follicles presented high degrees of GLUT-1 expression in second row, (IHC

Accordingly, the ovaries from VitE-received groups exhibited significantly (*p* < 0.05) higher amount of follicles with GLUT-1 positive stained follicular cells (Fig. 3).

**Fig. 4:**
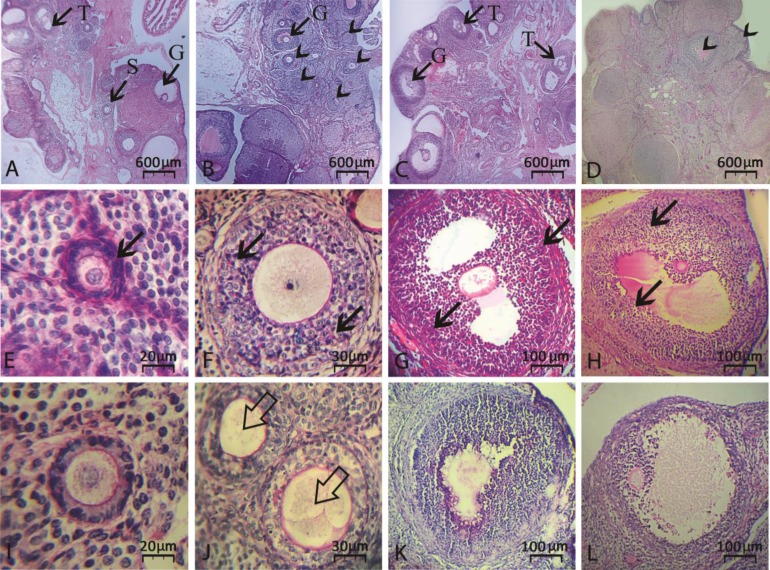
(**A**) Vitamin E-received group on day 14, see intact secondary (S), tertiary (T) and graafian (G) follicles (**B**) CPM alone-exposed group on day 14, (**C**) Vitamin E-received group on day 24 and (**D**) CMP alone-exposed group on day 24 after exposure. Note intact follicles from different stages in vitamin E-treated cross sections (arrows). Meanwhile, the cross sections from CPM alone-treated groups are presenting serious atresia (arrowheads), intact PAS reaction in primary (**E**), secondary (**F**), tertiary (**G**) and graafian (**H**) follicles are marked with arrows. However, atretic primary (**I**), secondary (**J**), tertiary (**K**) and graafian (**L**) follicles with significantly lower PAS reaction can be observed (PAS

**Fig. 5.**
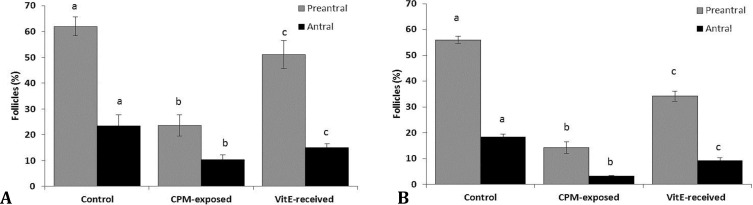
**A** **)** Mean percentage of follicles with moderate and intensive PAS reaction on day 14 and **B)** Mean percentage of follicles with moderate and intensive PAS reaction on day 24 after exposure to CPM, all data are presented as Mean ± SD.

^abc^ represent significant differences between marked data (*p* < 0.05).

The PAS staining was performed in order to estimate intracytoplasmic carbohydrate ratio. Similar to other findings, CPM reduced PAS reaction and VitE ameliorated the impairment. Accordingly, the percentage of follicles with PAS-stained follicular cells remarkably (*p* < 0.05) increased in VitE-received groups. Observations showed that VitE increases the distribution of intensive- and moderate-stained cells in VitE-received groups versus the CPM-exposed ones (Figs. 4 and Fig. 5). 

## Discussion

The present study showed that CPM by down-regulating the expression of GLUT-1 resulted in serious impairments in transferring of glucose through cell membrane and in turn it reduced the intact energy metabolism pathway(s). Therefore, follicular cells, especially granulosa cells, presented the high caspase-3 protein expression. However, more analyses showed that, VitE significantly reduced the CPM-induced detrimental impacts. Accordingly, the cross sections from VitE-received animals exhibited significantly higher follicular distribution at both preantral and antral stages. Moreover, the caspase-3 expression remarkably reduced in VitE-received animals versus CPM alone-exposed groups. 

Previously, we showed that CPM impacts on the follicular growth through down-regulating the angiogenesis, which is in corroboration with our new findings. It has been also clarified that angiogenesis as an essential demand is required for energy sources delivery through ovarian cortex.^27^ Indeed, the follicles at the late secondary and antral stages uptake the glucose directly via GLUTs and these follicles need glucose as main source for glycolysis and developing.^28^^,^^29^ Any deficiency in ovarian angiogenesis mainly affects the larger follicles growing mechanism by impairing the cellular signaling pathways as well as supplying essential energy sources. Here, we showed that lower size follicles exhibit decreased GLUT-1 expression at protein level which was not statistically considerable in larger ones. The last finding suggests that, small size follicles (< 300 micrometers) are more susceptible for GLUT-1 expression. Considering that theca cells layer are the unique area for angiogenesis,^27^^-^^29^ it would be more logic to conclude that larger size follicles (large and pre-ovulatory follicles) will be more susceptible to impaired angiogenesis than pie size and pre-antral ones. Thus, in energy dependent atresia, as it happens in CPM-exposed follicles, the follicular population is reduced in the ovaries by down-regulating angiogenesis at antral and grafian stages and reducing GLUT-1 expression at early pre-antral stages. However, co-administrating of VitE, resulted in a remarkable elevation in GLUT-1 protein biosynthesis. Moreover, our previous analyses showed that VitE considerably enhances the angio-genesis in CPM-induced ovaries.^8^ Thus, taking together, we can suggest that co-administration of VitE survives the preantral and antral follicles by up-regulating GLUT-1 expression and enhancing angiogenesis, respectively. 

On the other hand, association of oxidative stress as a parallel alteration should be considered beside CPM-induced direct cytotoxicity. We showed that chronic exposure to CPM significantly reduces the ovarian antioxidant status,^8^ which was corroborated with other reports in serum, liver and brain.^27^^,^^28^ Considering oxidative stress-induced RNA and DNA damages,^8^ normally it is expected that mRNA, and its consequence product (GLUT-1 protein) partly maintained/ protected against CPM-induced oxidative stress in VitE-received animals. However, FSH and IGF-1 co-association stimulates GLUT-1 expression at both protein and mRNA levels,^22^ suggesting that VitE indirectly enhances hypophysis endocrine status and ultimately provokes follicular cells proliferation/development by enhancing the GLUT-1 expression.

In order to understand how GLUT-1 affects the metabolism(s) of the follicular cells, one should note that intracytoplasmic glucose ratio individually is supplied via GLUTs.^21^^-^^23^ It is well established that diminished intra-cytoplasmic energy sources (mainly glucose) result in pro-apoptotic genes and proteins expression as well as biosynthesis, suggesting the vital role of GLUTs in inducing and/or stimulating apoptosis.^21^^,^^22^ Although caspase-3 is known for its protease reaction in provoking apoptosis, the main stimulatory factors for caspase-3 gene expression and biosynthesis are not completely understood. In present study we tried to uncover the relation between CMP-induced endocrine disruption and CPM-decreased GLUT-1 expression with caspase-3. Our histological and computer-assessed software data showed that CPM resulted in an intensive expression of caspase-3 in follicular and stromal cells of the ovaries. Gonadotropins actually are involved in stabilizing the pro-apoptotic gene expression as well as protein synthesis by inhibiting involving genes expression and even translocation.^15^^,^^17^ It has been reported that CPM exposure results in reduction of FSH and LH levels.^27^ Therefore, CPM likely by reducing the gonadotropins synthesis, triggered the caspase-3 expression. However, the roles of oxidative stress beside decreased GLUT-1 synthesis (in line with energy dependent apoptosis) should not be excluded. Indeed, irreparable DNA, RNA and nucleotides damages result in protease interaction and highly expression of caspase-3 in granulosa cells.^27^^-^^29^ Therefore, reduced biosynthesis of caspase-3 protein in VitE-received animals may be attributed to its potential antioxidant property, which protects the cellular DNA and RNA content. On the other hand, VitE may partially diminish the caspase-3 and/or other pro-apoptotic stimulators expression/biosynthesis via up-regulating the gonadotropins secretion. Albeit, this hypothesis has shown by Pan *et al*. for VitE in earlier studies.^30^

In conclusion, our data showed that, CPM resulted in a significant decrease in GLUT-1 expression, which in turn resulted in a remarkable reduction in intracytoplasmic carbohydrate ratio. However, VitE by recovering the GLUT-1 expression enhanced the glucose transport. Moreover, we showed that reduced main energy source results in accelerated caspase-3 biosynthesis that elevates apoptosis in follicular cells and oocyte levels. Meanwhile, VitE by improving the antioxidant status and carbohydrate transformation reduced CPM-induced apoptosis.

## References

[1] Usmani KA, Knowles CO (2001). Toxicity of pyrethroids and effect of synergists to larval and adult Helicoverpa zea, Spodoptera frugiperda, and Agrotis ipsilon (Lepidoptera: Noctuidae). J Econ Entomol.

[2] Kojima H, Katsura E, Takeuchi S (2004). Screening for estrogen and androgen receptor activities in 200 pesticides by in vitro reporter gene assays using Chinese hamster ovary cells. Environ Health Perspect.

[3] Wilson VS, Bobseine K, Gray LE Jr (2004). Development and characterization of a cell line that stably expresses an estrogen-responsive luciferase reporter for the detection of estrogen receptor agonist and antagonists. Toxicol Sci.

[4] Xia Y, Bian Q, Xu L (2004). Genotoxic effects on human spermatozoa among pesticide factory workers exposed to fenvalerate. Toxicology.

[5] Xu LC, Zhan NY, Liu R (2004). Joint action of phoxim and fenvalerate on reproduction in male rats. Asian J Androl.

[6] Song L, Wang YB, Sun H (2008). Effects of fenvalerate and cypermethrin on rat sperm motility patterns in vitro as measured by computer-assisted sperm analysis. J Toxicol Environ Health A.

[7] Krieger RI (2001). Handbook of pesticide toxicology.

[8] Molavi M, Razi M, Malekinejad H (2014). Vitamin E improved cypermethrin-induced damages in the ovary of rats; Evidence for angiogenesis and p53 involvement. Pestic Biochem Physiol.

[9] Yoshida Y, Niki E, Noguchi N (2003). Comparative study on the action of tocopherols and tocotrienols as antioxidant: chemical and physical effects. Chem Phys Lipids.

[10] Erin AN, Spirin MM, Tabidze LV (1984). Formation of α-tocopherol complexes with fatty acids. A hypothetical mechanism of stabilization of biomembranes by vitamin E. Biochim Biophys Acta.

[11] Jain SK (1983). Vitamin E and stabilization of membrane lipid organization in red blood cells with peroxidative damage. Biomed Biochim Acta.

[12] Lucy J (1972). Functional and structural aspects of biological membranes: A suggested structural role for vitamin E in the control of membrane permeability and stability. Ann N Y Acad Sci.

[13] Mun JY, Lee WY, Han SS (2005). Effects of cypermethrin on the dopaminergic neurons in the progressive hemi-parkinsonian rats. Toxicol Mech Methods.

[14] Akhtari K, Razi M, Malekinejad H (2012). Uterine artery interruption: Evidence for follicular growth and histochemical and biochemical changes. J Reprod Infertil.

[15] Tilly JL, Kowalski KI, Johnson AL (1991). Involvement of apoptosis in ovarian follicular atresia and post-ovulatory regression. Endocrinology.

[16] Yacobi K, Wojtowicz A, Tsafriri A (2004). Gonadotropins enhance caspase-3 and-7 activity and apoptosis in the theca-interstitial cells of rat pre-ovulatory follicles in culture. Endocrinology.

[17] Johnson AL (2003). Intracellular mechanisms regulating cell survival in ovarian follicles. Anim Reprod Sci.

[18] Jänicke RU, Ng P, Sprengart ML (1998). Caspase-3 is required for α-fodrin cleavage but dispensable for cleavage of other death substrates in apoptosis. J Biol Chem.

[19] Wood IS, Trayhurn P (2003). Glucose transporters (GLUT and SGLT): Expanded families of sugar transport proteins. Br J Nutr.

[20] Williams S, Blache D, Martin GB (2001). Effect of nutritional supplementation on quantities of glucose transporters 1 and 4 in sheep granulosa and theca cells. Reproduction.

[21] Kol S, Ben-Shlomo I, Ruutiainen K (1997). The midcycle increase in ovarian glucose uptake is associated with enhanced expression of glucose transporter 3. Possible role for interleukin-1, a putative intermediary in the ovulatory process. J Clin Invest.

[22] Kodaman PH, Behrman HR (1999). Hormone-regulated and glucose-sensitive transport of dehydroascorbic acid in immature rat granulosa cells. Endocrinology.

[23] Zhou J, Bievre M, Bondy CA (2000). Reduced GLUT1 expression in Igf1–/–null oocytes and follicles. Growth Horm IGF Res.

[24] Zhang C, Niu W, Wang Z (2012). The effect of gonadotropin on glucose transport and apoptosis in rat ovary. PloS One.

[25] Yousef MI, Abdallah GA, Kamel KI (2003). Effect of ascorbic acid and vitamin E supplementation on semen quality and biochemical parameters of male rabbits. Anim Reprod Sci.

[26] North PE, Waner M, Mizeracki A (2000). GLUT1: A newly discovered immunohistochemical marker for juvenile hemangiomas. Hum Pathol.

[27] Ateşşahin A, Yilmaz S, Karahan I (2005). The effects of vitamin E and selenium on cypermethrin-induced oxidative stress in rats. Turk J Vet Anim Sci.

[28] Sankar P, Telang AG, Manimaran A (2012). Protective effect of curcumin on cypermethrin-induced oxidative stress in Wistar rats. Exp Toxicol Pathol.

[29] Giray B, Gürbay A, Hincal F (2001). Cypermethrin-induced oxidative stress in rat brain and liver is prevented by vitamin E or allopurinol. Toxicol lett.

[30] Pan SY, van Dyke HB, Kaunitz H (1949). Effect of vitamin -E deficiency on amount of gonadotropin in the anterior pituitary of rats. Exp Biol and Med.

